# A case report of fungemia due to *Kodamaea ohmeri*

**DOI:** 10.1186/s12879-019-4208-8

**Published:** 2019-07-01

**Authors:** K. Diallo, B. Lefevre, G. Cadelis, J. C. Gallois, F. Gandon, M. Nicolas, B. Hoen

**Affiliations:** 1 Department of Infective and Tropical Diseases, Dermatology and Internal Medicine, University Hospital of Pointe à Pitre, Pointe à Pitre, France; 2 Lung diseases unit, University Hospital of Pointe à Pitre, Pointe à Pitre, France; 3 Laboratory of bacteriology, parasitology and mycology, University Hospital of Pointe à Pitre, Pointe à Pitre, France; 4 INSERM Clinical Investigation Center 1424, University Hospital of Pointe à Pitre, Pointe à Pitre, France; 5 University of the French West Indies and French Guyana, Medical School Hyacinthe Bastaraud, EA 4537 Pointe à Pitre, France

**Keywords:** *Kodamaea ohmeri*, Fungemia

## Abstract

**Background:**

*Kodamaea ohmeri* is a yeast is frequently mistaken for *Candida,* which belongs to the same family. This micro-organism has been reported to cause life-threatening infections in humans.

**Case presentation:**

A 81-year-old woman developed a severe fungemic pulmonary infection due to *Kodamaea ohmeri* that was identified from bronchoalveolar fluid and blood cultures, which is unusual in immunocompetent patients. Because *K. ohmeri* was first wrongly identified as *Candida albicans*, the patient inadequately received caspofungin, which was clinically ineffective, especially as the strain was resistant to echinocandins. Clinical cure was obtained after treatment was switched to voriconazole.

**Conclusions:**

An increasing number of serious infections due to *K. ohmeri* has been reported in the literature, but the correct identification of this micro-organism remains difficult.

## Background

*Kodamaea ohmeri*, previously known as *Pichia ohmeri*, belongs to the *Ascomycetae* class and the *Saccharomycetaceae* family. It is the teleomorphic form of *Candida guilliermondii* [1]. This yeast is frequently mistaken for *Candida,* which belongs to the same family [2]. It has been first described in tree barks, fruits and cucumber salts used in the food industry for the fermentation of pickled foods. It has also been isolated from environmental sources such as pools, sand, floors and sea water [2]. Since 1984, this micro-organism has been reported to cause life-threatening infections in humans. An increasing number of serious infections has been reported in the literature. We herein report on a new case.

## Case presentation

A 81-year-old woman was admitted to the emergency room on January 19th, 2018 for fever and vomiting. Her medical history consisted only in mild cognitive disorders and she received no treatment. Her temperature was 39.2 °C, the oxygen saturation while breathing room air was 88%, and clinical examination was remarkable for rhonchi, extracellular dehydration, fecal impaction, and poor oral condition. Total white blood cell count was 12.7 G/L (PMN 11.2 G/L), serum creatinine, sodium and calcium were 179 μmol/L, 149 mmol/L, and 2.04 mmol/L, respectively. Serum C-reactive protein was 613 mg/L. Liver and pancreatic parameters were normal. Serum CPK and LDH levels were 490 IU/L and 1239 IU/L, respectively. Total body CT-scan showed bilateral basal pulmonary condensations associated with interstitial infiltrates in the upper lobes, as well as an excavated condensation in the right upper lobe and non-complicated colonic diverticulosis. Amoxicillin-clavulanate was started on an empirical basis and the patient was admitted to the pneumology department. Sputum smears were repeatedly negative for acid-fast bacilli. Several blood cultures drawn within the first 3 days remained negative. Urinalysis was negative as were antigenuria for *Legionella pneumophila* and *Streptococcus pneumoniae*. Serologies were negative for HIV, HCV, and HTLV-1/2 and positive for anti-HBs antibodies. Because of persisting fever after 10 days of antibiotic treatment, a bronchoscopy was performed, which found diffuse bronchomalacia and no visible tumor. Lavage fluid culture was positive for *Staphylococcus aureus* (10^4^ CFU/mL) and *Candida* spp. (10^3^ CFU/mL). The latter contained two populations that were first identified as *C. albicans* and a *C. parapsilosis*. The infection was not catheter related, and no other primary focus was identified. Oral fluconazole was started on February 1st. On February 6, while the patient remained febrile, an aerobic blood culture was positive for a yeast after 28 h of growth. Intravenous caspofungin was started. The yeast was identified as *K. ohmeri* with API 20C System, which led to re-identify the strain of *Candida albicans* that had been cultured from the BAL fluid as *K. ohmeri.* With E-test method, the strain was found to be resistant to fluconazole (MIC 4 mg/L) and caspofungin (MIC > 2 mg/L) but sensitive to voriconazole (MIC 0.05 mg/L) and amphotericin B (MIC 0.09 mg/L). Antifungal therapy was switched to voriconazole 4 mg/kg/day. All subsequent blood cultures were negative. Transthoracic echocardiography showed no image consistent with endocarditis. The patient became afebrile and was weaned from oxygen within a few days and voriconazole was discontinued after 10 days. The treatment was stopped by the physicians while the outcome was favourable.

## Discussion

This patient presented with a fungemic infection due to *K. ohmeri*. The outcome was favorable after introduction of voriconazole.

*K. ohmeri* is a yeast known to cause invasive infections [3]. In the literature, we identified 73 cases of fungemia due to *K. ohmeri* [2, 4, 5], 2 cases of peritonitis [6, 7], 3 cases of endocarditis [8–10], one case of urinary tract infection [11], one case of cellulitis [12].

In most of these cases, patients were immunosuppressed because of hematologic or solid malignancies, post-chemotherapy neutropenia, immunosuppressive treatments, diabetes, or chronic renal failure [13]. Many cases were catheter-related, and catheter removal was key to cure. Pre-exposure to systemic antibiotics was also reported in many cases [4]. Systemic infections due to *K. ohmeri* are rarely reported in immunocompetent subjects [14].

On solid media, *K. ohmeri* forms Candida-like colonies and change their color from pink to blue within 48 h on CHROMagar as do *Candida* spp. which explains why *K. ohmeri* may often be mistaken for *Candida* spp*. K. ohmeri* is usually resistant to fluconazole and sensitive to amphotericin B [15]. It is also usually sensitive to echinocandins [13], which was not the case in our case.

According to the Clinical Practice Guideline for the Management of Candidiasis of the Infectious Diseases Society of America, an echinocandin is recommended as initial therapy [16]. Fluconazole, 800 mg (12 mg/kg) loading dose, then 400 mg (6 mg/kg) daily, is an alternative for patients who are not critically ill and have had no prior exposure to azoles. In our case, none of these first-line treatments used for candidemia were effective.

## Data Availability

Not applicable.
